# Parasitosis by *Fasciola hepatica* and Variations in Gut Microbiota in School-Aged Children from Peru

**DOI:** 10.3390/microorganisms12020371

**Published:** 2024-02-11

**Authors:** Wilmer Silva-Caso, Hugo Carrillo-Ng, Miguel Angel Aguilar-Luis, Yordi Tarazona-Castro, Luis J. Del Valle, Carmen Tinco-Valdez, Carlos Palomares-Reyes, Numan Urteaga, Jorge Bazán-Mayra, Juana del Valle-Mendoza

**Affiliations:** 1 Research and Innovation Centre, Faculty of Health Sciences, Universidad Peruana de Ciencias Aplicadas, Lima 15023, Peru; hugo.carrillo.n@upch.pe (H.C.-N.); miguel.aguilar@upc.pe (M.A.A.-L.); pcmeytar@upc.edu.pe (Y.T.-C.); carmen.tinco@carloscueto.edu.pe (C.T.-V.); pcmecpal@upc.edu.pe (C.P.-R.); 2 Instituto de Investigación Nutricional, Lima 15024, Peru; 3 Escuela de Medicina, Facultad de Ciencias de la Salud, Universidad Peruana de Ciencias Aplicadas, Lima 15023, Peru; 4 Barcelona Research Center for Multiscale Science and Engineering, Departament d’Enginyeria Química, EEBE, Universitat Politècnica de Catalunya (UPC), 08019 Barcelona, Spain; luis.javier.del.valle@upc.edu; 5 Puesto de Salud Callancas, Dirección Regional de Salud Cajamarca (DIRESA), Cajamarca 60101, Peru; numan1030@gmail.com; 6 Laboratorio Regional de Cajamarca, Dirección Regional de Salud de Cajamarca (DIRESA), Cajamarca 60101, Peru; jbazanm@diresacajamarca.gob.pe

**Keywords:** fascioliasis, gut microbiota, dysbiosis

## Abstract

(1) Background: Human fascioliasis is considered an endemic and hyper-endemic disease in the Peruvian Andean valleys. Our objective was to determine variations in the composition of the gut microbiota among children with *Fasciola hepatica* and children who do not have this parasitosis. (2) Method: A secondary analysis was performed using fecal samples stored in our biobank. The samples were collected as part of an epidemiological *Fasciola hepatica* cross-sectional study in children from 4 through 14 years old from a community in Cajamarca, Peru. (3) Results: In a comparison of the bacterial genera that make up the intestinal microbiota between the *F. hepatica* positive and negative groups, it was found that there are significant differences in the determination of *Lactobacillus* (*p* = 0.010, CI: 8.5–61.4), *Bacteroides* (*p* = 0.020, CI: 18.5–61.4), *Clostridium* (*p* < 0.001, CI: 3.5–36.0), and *Bifidobacterium* (*p* = 0.018, CI: 1.1–28.3), with each of these genera being less frequent in children parasitized with *F. hepatica*. (4) Conclusions: These results show that *F. hepatica* may be associated with direct or indirect changes in the bacterial population of the intestinal microbiota, particularly affecting three bacterial genera.

## 1. Introduction

*Fasciola hepatica* is a food-borne trematode with worldwide distribution that causes infections in humans and animals [1]. Fascioliasis causes around 17 million cases, and 180 million people are at risk of infection worldwide, predominantly in resource-limited countries [1,2]. It was considered primarily a disease of ruminants; however, over the past two decades, human fascioliasis has gained significance as an important public health problem [3]. Moreover, the World Health Organization (WHO) now includes this disease in the list of the most important neglected tropical diseases (NTDs).

Human fascioliasis is considered an endemic and hyper-endemic disease in the Peruvian Andean valleys, particularly in areas where livestock breeding and freshwater plant ingestion are common [4,5]. However, many cases may remain underdiagnosed, as approximately 50% of human infections are asymptomatic [1]. The natural history of *Fasciola hepatica* infection is characterized by an acute phase, characterized by the migration of the juvenile parasite through the intestinal wall to the liver parenchyma, and a chronic phase in which mature parasites obstruct and cause biliary tract inflammation [3,4,5]. During both stages, the disease causes a large impact on human health owing to its high pathogenicity and immune modulation [2,6,7].

The treatment of fascioliasis is anthelmintic therapy. The drug of choice is triclabendazole, which has a cure rate greater than 90%. This drug has also been used in children under 6 years of age. The alternative option is nitazoxanide, although evidence is limited to observational studies [8,9]. Additional intervention may be needed depending on the nature of the clinical presentation. In these cases, biliary decompression (endoscopic, percutaneous, or surgical) may be justified [10,11].

The gut microbiota is now considered an “essential organ”, which possesses 150 times more genes than the ones found in the entire human genome, with symbiotic, physiological, and pathophysiological implications [12,13,14]. This bacterial community plays a pivotal role in human homeostasis and is involved in the extraction of nutrients from food, the maturation of the intestinal mucosa, immune-system development, and protection against opportunistic pathogens [12,14].

Infectious diseases are among the most common causes of imbalances in the composition and function of the microbiota, which are also known as dysbiosis [13,15]. Most reports studied the relationship between bacterial infections and gut microbiota. However, the interaction between helminths and gut microbiota has recently gained research interest and speculation in terms of host health [15]. There is a three-way interaction between the host, helminths, and the gut microbiome, which has a great influence on host health [15,16]. For example, both helminths and gut bacteria modulate the host’s immune system, reducing inflammatory responses directed against themselves and unrelated antigens [16,17].

Some studies in humans and animals have shown that parasitic infections cause changes in the composition and abundance of the microbiota [18,19,20,21,22]. This is because the animal intestine is a highly complex and polymicrobial ecosystem, with the intestinal microbiota affecting the entire host organism at different levels. For example, the microbiota is related to the regulation of lipid storage and the stimulation or renewal of epithelial cells, and it has an influence on the development and maturation of the brain and immune system. The microbiota have been described as playing a role in protection against infection by pathogenic bacteria, viruses, fungi, and parasites. Thus, maintaining homeostasis between the intestinal microbiota and the rest of the body is essential for health. However, the mechanisms that govern the interaction between the intestinal microbiota and parasites have not been clarified. Relevant information on the subject indicates that the composition of intestinal bacterial populations modulates the progression of infection by parasites, especially protozoa, while also modulating the outcome of the parasitic disease, which highlights the protective role of the microbiota against various infectious processes. In this context, the possible consequences of microbiota alterations in parasitic, allergic, and autoimmune disorders have also been studied [23]. Despite the above, the effect of *F. hepatica* infection on the human intestinal microbiota has not yet been studied. It is crucial to understand the mechanisms and pathways involved in the three-way interaction between the host individual, parasitic microorganisms, and the intestinal microbiota. In this context, the objective of this study was to identify variations in the composition of the intestinal microbiota of school-aged children with and without *F. hepatica* infection.

## 2. Materials and Methods

### 2.1. Patients and Sampling

A secondary analysis was performed using fecal samples stored in our biobank. The samples were collected as part of an epidemiological *Fasciola hepatica* cross-sectional study, which was conducted from April to June 2015 in children from 4 to 14 years old from a community in San Pablo (Cajamarca, Peru). The inclusion criteria were children of school age who lived in the study area for at least 6 months. Children who received antiparasitic treatment or laxatives in the 15 days prior to sample collection and individuals with other known parasitic infections were excluded from the study. The population studied was homogeneous, which reduces factors that could interfere with the interpretation of the results when analyzing the gut microbiota.

### 2.2. Ethics Statement

This study was approved by the Research Ethics Board of the Hospital Regional Docente de Cajamarca. The samples were obtained in the context of the epidemiological surveillance program of the DIRESA-Cajamarca through the comprehensive surveillance network and the specialized information system for the disease.

### 2.3. Samples and Sample Processing

#### 2.3.1. Samples

Fecal samples were collected by the children’s caregivers in a sterile container and stored at −20 °C until they were processed. The data for each patient were obtained using an epidemiological questionnaire designed by the Peruvian Ministry of Health.

#### 2.3.2. Detection of *Fasciola hepatica* by ELISA

Presence of *F. hepatica* was determined by ELISA using the commercial Bio-X *Fasciola hepatica* antigenic ELISA kit (Bio-X diagnostics, Rochefort, Belgium), according to manufacturer’s instructions. This kit is designed to detect excretory fecal antigens, based on the MM3-COPRO ELISA test. It is the only available commercial version of the aforementioned assay and has been validated as an effective mass screening tool in the field [24].

#### 2.3.3. DNA Extraction

DNA was extracted from the sample aliquots using the innuPREP DNA stool kit (Analytik Jena, Jena, Germany), according to the manufacturer’s instructions. Extracted DNA was then eluted in 100 µL nuclease-free buffer and stored at −80 °C until use.

#### 2.3.4. PCR Amplification for Detection of Gut Microbiota

We evaluated the presence of 13 representative bacteria genera in gut microbiota. The genera of bacteria analyzed were *Actinobacteria*, *Bacteroides*, *Bacteroidetes*, *Bifidobacterium*, *Clostridium*, *Enterococcus*, *Eubacterium*, *Firmicutes*, *Fusobacterium*, *Lactobacillus*, *Prevotella*, *Proteobacteria*, and *Veillonella*. The PCR was performed using primers and conditions described previously [25,26]. Specific primers targeting different bacterial genera were used to characterize the microbiota in fecal samples using real-time PCR. All PCR tests were carried out in duplicate, with a final volume of 20 μL. The time and temperature conditions used were the following: initial denaturation of the DNA at 95 °C for 10 min; 45 cycles of denaturation at 95 °C for 10 s; hybridization or annealing at optimal temperature for 20 s; and extension at 72 °C for 15 s. DNA extracted from each participant was subjected to human β-globin PCR to ensure that amplifiable DNA was successfully extracted from the sample and to control for PCR inhibitors with the described reaction conditions. In each analysis, negative controls containing all elements of the reaction mixture except the extracted DNA were performed, and no amplified product was detected under these conditions [25].

### 2.4. Statistical Analysis

The database was managed using Microsoft Excel, the statistical analysis was performed using Minitab18, and the graphics were created using OriginPro v10 software. Qualitative variables were reported as frequencies and percentages. A *t*-test or Kruskal–Wallis test was applied to evaluate differences for continuous variables. Categorical variables were assessed using χ^2^ test and Fisher’s exact test. Values of *p* < 0.05 were considered significant.

## 3. Results

The study participants were divided into two groups: those who had a negative antigen test for *F. hepatica* by ELISA and those who tested positive for the pathogen. No differences were found by age group between 4 to 8 years and 9 to 14 years. Nor were any differences found between parasitized and non-parasitized according to gender (Table 1).

In the analysis of the risk factors for contracting *F. hepatica*, no differences were found between both groups in reference to the drinks and foods ingested and being in contact with livestock (Table 2).

The clinical symptoms related to parasitosis that were present in both study groups were evaluated, such as abdominal pain in the last 3 months, weight loss in the last 3 months, diarrhea in the last 3 months, headache, nausea, vomiting, fever, and abdominal pain. For each of these clinical characteristics, no differences were found between *the F. hepatica* positive and negative groups. However, a higher frequency of vomiting and diarrhea was observed in the last 3 months in the group parasitized by *F. hepatica* (Table 3).

**Figure 1:**
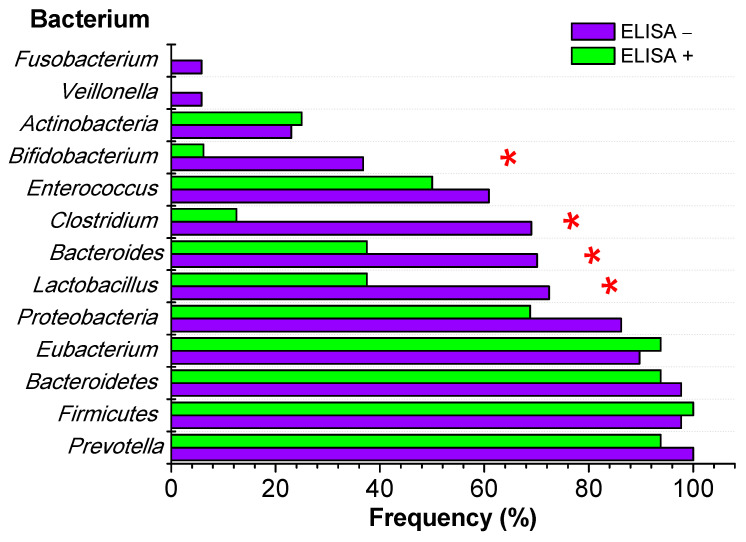
Gut microbiota composition according to *F. hepatica* infection diagnosed by ELISA. * *p* < 0.05, Fisher’s exact test.

In a comparison of the bacterial genera that make up the intestinal microbiota between the *F. hepatica* positive and negative groups, it was found that there are significant differences in the determination of *Lactobacillus* (*p* = 0.010, CI: 8.5–61.4), *Bacteroides* (*p* = 0.020, CI: 18.5–61.4), *Clostridium* (*p* < 0.001, CI: 3.5–36.0), and *Bifidobacterium* (*p* = 0.018, CI: 1.1–28.3), with each of these genera being less frequent in children parasitized with *F. hepatica*. In the other nine bacterial genera evaluated, *Prevotella*, *Firmicutes*, *Bacteroidetes*, *Eubacterium*, *Proteobacteria*, *Enterococcus*, *Actinobacteria*, *Veillonella*, and *Fusobacterium*, no differences were found between the studied groups (Figure 1, Table 4).

## 4. Discussion

Nowadays, it is important to understand the interaction between parasites, the intestinal microbiota and their effect on the host’s health. In this context, there are no studies that analyze *F. hepatica* parasitosis and its impact on the intestinal microbiota in children. Firstly, the participants were divided into two groups according to the presence or absence of parasitosis by this microorganism, and their demographic and clinical characteristics were evaluated without observing significant variations between both groups. Previous studies also did not find significant differences between 36 analyzed variables, including demographic data and clinical symptoms, between groups of parasitized and non-parasitized children [27].

In our study, children with fascioliasis showed a lower frequency in the identification of *Bacteroides*, *Bifidobacterium*, *Lactobacillus*, and *Clostridium* compared with children who tested negative for this parasitosis. These findings suggest that the presence of this parasite may be associated with variations in the microbiota, particularly affecting the number of beneficial commensal bacteria. There are several mechanisms by which helminths can alter the intestinal microbiota, either through direct or indirect interaction. For example, during its biological cycle, *Fasciola* penetrates the intestinal mucosa to migrate toward the liver parenchyma, which in turn can affect the bacterial population residing in the intestine [1]. It was reported that helminths can alter mucus production and epithelial permeability, producing changes in the microbiota [17].

Furthermore, it was described that *F. hepatica* can modulate the immune system and its response against different antigens, including those of the resident bacteria that make up the intestinal microbiota [15,16,17]. It was also shown that this parasite can affect inflammasome activation and consequently cause microbial dysbiosis [17,28,29]. This behavior may be due to interactions between the gut microbiota and liver disease caused by *F. hepatica*. In this context, it is known that some hepatic metabolites, such as bile acids, influence the microbial composition of the intestine through the hepatic portal system and bile secretion systems [30,31]. The imbalance of the intestinal microbiota was described in patients with liver disease caused, for example, by the hepatitis B virus (HBV), where an increase in bacterial translocation and the presence of endotoxins at the level of the portal vein are described. This would explain the activation of the Toll-like receptor (TLR) and the NOD-like receptor (NLR) in the liver. Both activated TLR-NLR receptors would enhance a general inflammatory response in the host, dependent on pro-inflammatory signal transduction pathways such as those mediated by nuclear factor kappa B (NF-κB). At the same time, the production and secretion of pro-inflammatory cytokines are accelerated, such as tumor necrosis factor alpha (TNF-α), which is involved in chronic inflammation processes and leads to the development of liver lesions [31,32,33,34]. This would partly explain why individuals who develop the disease present with liver lesions during ultrasound examination, eosinophilia in hematological examinations, and variations in the intestinal microbiota [35]. Regarding fascioliasis and liver lesions, migrating metacercariae cause the destruction of the liver parenchyma, causing necrosis and fibrosis. In addition, adult trematodes can obstruct the bile ducts, resulting in thickening, dilation, and fibrosis of the proximal biliary tree, as they reside in the host bile ducts [36]. Liver damage is correlated with the parasite load and the lifespan of adult *F. hepatica* trematodes, which in humans is 9 to 13 years. The infestation has two phases. In the acute phase, metacercaria migration through the liver is associated with fever, right upper-quadrant pain, and hepatomegaly. This phase can be complicated by hemobilia or subcapsular hematomas of the liver [37]. In very severe infections, extensive necrosis of the liver parenchyma may occur. In the chronic phase, obstruction of the common bile duct can develop, causing biliary colic, cholangitis, cholelithiasis, and obstructive jaundice. A prolonged and/or severe infection can also cause sclerosing cholangitis and biliary cirrhosis [38,39].

Our results show that the frequency of beneficial bacteria such as *Bacteroides*, *Bifidobacterium*, *Lactobacillus*, and *Clostridium* decreased in infected children. These bacteria are commensal microorganisms that play an essential role in host intestinal homeostasis and the development of the immune system, specifically the gut-associated lymphoid tissue (GALT) [40]. The colonization of germ-free mice with the genus *Bacteroides* improved their underdeveloped intestinal immune system, producing an increase in lymphoid tissue in Preyer’s plaques and activating the T cell-related immune response [41]. Furthermore, *Bacteroides* plays a role in preventing specific *Clostridium* difficile infections [41,42]. Commensals *Lactobacillus* and *Bifidobacterium* maintain intestinal homeostasis and exhibit a protective role against irritable bowel disease and inflammatory bowel disease [43]. Both have been used as probiotics because they can inhibit the growth of harmful bacteria, improve gastrointestinal barrier function, and suppress the production and release of pro-inflammatory cytokines [43,44]. The genus of commensal bacteria *Clostridium* has multiple important metabolic functions through the release of butyrate, which is an essential metabolite for colonocytes [45]. Indeed, butyrate inhibits the activation of the transcription factor NF-kB, leading to a decrease in the expression of proinflammatory cytokines [46,47]. It also demonstrates a protective effect against colitis and colorectal cancer, showing an apoptotic effect on tumor cells in vitro [47]. Due to the above, the decrease in these bacterial populations would have an unfavorable impact on the health of the population. There are no published human studies that provide estimates of the impact of the liver worm *Fasciola hepatica* on the composition and relative abundance of intestinal microbial communities. However, a study carried out in non-human mammals reported that *F. hepatica* reduced the diversity in both prokaryotic and eukaryotic microorganisms of the intestinal microbiota. Ramírez et al. highlighted that the composition of the intestinal microbiota is affected by other factors in addition to the studied variable, such as diet, age, geographical location, and previous diseases [48,49]. By taking these considerations into account, we highlight the homogeneous characteristics in age, diet, and geographical location of our population. Furthermore, since these were children between 4 and 14 years of age, chronic diseases were not considered as a factor that could alter the composition of the intestinal microbiota, such as type 2 diabetes mellitus [50].

With regard to the impact of the infection caused by other parasites on the bacterial genera analyzed in our research, there is increasing evidence that parasitic infections have a detrimental effect on the intestinal microbiota. For example, it was decribed that parasitosis by *Trichuris muris* in mice was associated with a reduction in the diversity and abundance of *Bacteroidetes* and *Prevotella*, with attenuated assimilation of carbohydrates and a reduction in vitamin D derivatives [18]. Furthermore, the presence of *Blastocystis* was associated with a significant decrease in protective bacteria such as *Bifidobacterium* in patients with irritable bowel disease [20]. Children infected with *Schistosoma hemaetobium*, another foodborne trematode, showed a higher abundance of bacteria than healthy children, with *Bacteroidetes*, *Firmicutes*, and *Proteobacteria* being the most abundant phyla [21].

The studies we reviewed on the impact of parasitosis on the intestinal microbiota focused on the fact that the parasite needs a host to survive so it interacts and influences the microbiota. The parasite involved in microbiota alterations can be an intestinal parasite or a liver parasite. This is how *Blastocystis* is associated with changes in the composition of the intestinal microbiota; these changes have the *Firmicutes–Bacteroidetes* relationship as an indicator. On the other hand, it was found that the *Bifidobacterium* genus was significantly reduced in patients with both irritable bowel syndrome and *Blastocystis*, in addition to a significant decrease *in Faecalibacterium prausnitzii*, which has anti-inflammatory properties in *Blastocystis* infection. It was also reported that *Lactobacillus* species are involved in reducing the presence of *Giardia*, and the bacteriocins produced prevent the adhesion of the parasite. The presence of helminths, in particular as intestinal parasites, has been strongly associated with the transition from *Bacteroidetes* to *Firmicutes* and *Clostridia*. However, we report that there are no changes in *Bacteroidetes* between individuals parasitized and non-parasitized by *Fasciola hepatica*. In this context, microbiota studies have recently gained importance, and it is expected that they will contribute in the future to the understanding and management of various diseases as well as the fight against parasitic diseases [51].

Based on what was mentioned in the studies reviewed and according to our results, it is worth highlighting that helminth infections are recognized as a growing public health problem. This consideration covers Latin America and worldwide, especially developing countries where there is a lack of hygienic conditions due to socioeconomic factors that often imply a precarious quality of life in the population. Although published reports on the relationship between helminths and intestinal microbiota focused on parasites that have tropism for the intestines, we report a helminth with liver tropism that can alter intestinal physiology and other cellular parameters such as permeability, mucus secretion, and the production of antimicrobial peptides that cause dysbiosis. These microorganisms, in fact, survive among different commensal bacterial populations and can have an effect on altering the structure of the intestinal microbiota. Therefore, it is suggested that helminth infection influences the total number of bacterial species observed in the intestines of children [17,51].

## 5. Conclusions

We performed the first study examining the impact of *F. hepatica* parasitosis in the presence of 13 genera of bacteria representative of the intestinal microbiota. These results show that *F. hepatica* may be associated with direct or indirect changes in the bacterial population of the intestinal microbiota, particularly affecting three bacterial genera. More studies are needed to elucidate the complex relationship between *F. hepatica* and the intestinal microbiota and its effect on host health. Considering that these microorganisms affect the microbiome of the mammalian host in a species-specific way, therein lies the importance of our research.

### Limitations

A limitation of this study is that given the nature of the study design, we could not determine whether the infection by *F. hepatica* causes gut microbiota dysbiosis or if certain microbiota profiles make subjects more susceptible to infection by this parasite. Another limitation is that we did not perform quantification of the bacteria and only determined the presence or absence of certain bacteria. Finally, it was reported that infection by *F. hepatica* is usually accompanied by coinfection by other helminths; therefore, the roles of other concomitant infections need to be elucidated.

## Figures and Tables

**Table 1:** Frequency of *F. hepatica* infection diagnosed by ELISA in school-aged children from San Pablo, Cajamarca, according to age and gender.

Characteristics	Total	ELISA−	ELISA+		Odds Ratio	Fisher Exact Test *p*
	Frequency N = 103 (%)	Frequency N = 87 (%)	Frequency N = 16 (%)	95% CI		
Age (range years)						
4–8	45 (43.7)	38 (43.7)	7 (43.8)	23.1–66.8	1.003	1.000
9–14	58 (56.3)	49 (56.3)	9 (56.2)	33.2–76.9	1.000	1.000
Gender						
Female	48 (46.6)	41 (47.1)	7 (43.8)	23.1–66.8	0.873	1.000
Male	55 (53.4)	46 (52.9)	9 (56.2)	33.2–76.9	1.146	1.000

**Table 2:** Risk factors for *F. hepatica* infection in school-aged children from San Pablo, Cajamarca.

Risk Factors		Total	ELISA−	ELISA+		Odds Ratio	Fisher Exact Test *p*
		Frequency N = 103 (%)	Frequency N = 87 (%)	Frequency N = 16 (%)	95% CI		
Drinks	Boiled water	80 (77.7)	69 (79.3)	11 (78.6)	44.4–85.8	0.574	0.344
	Non-boiled water	72 (69.9)	58 (66.7)	14 (87.5)	64.0–96.5	3.500	0.139
	Emollients/infusions	33 (32.0)	32 (36.8)	1 (6.2)	1.1–28.3	0.115	0.018
	Ditch water	18 (17.5)	11 (12.6)	7 (43.8)	23.1–66.8	5.374	0.007
Meal	Andean lupin	99 (96.1)	84 (96.6)	15 (93.8)	71.7–98.9	0.536	0.497
	Raw lettuce	96 (93.2)	81 (93.1)	15 (93.8)	71.7–98.9	1.111	1.000
	Raw salad	84 (81.6)	79 (90.8)	15 (93.8)	71.7–98.9	1.519	1.000
	Watercress	34 (33.0)	31 (35.6)	3 (18.8)	6.6–43.0	0.417	0.253
	Chews grass	10 (9.7)	10 (11.5)	0 (0.0%)	0.0–19.4	0.000	0.355
Livestock contact	Yes	95 (92.2)	80 (92.0)	15 (93.8)	71.7–98.9	1.312	1.000
	No	8 (7.8)	7 (8.0)	1 (6.2)	1.1–28.3	0.762	1.000

**Table 3:** Clinical symptoms associated with *F. hepatica* infection diagnosed by ELISA in school-aged children from San Pablo, Cajamarca.

Clinical Symptoms	ELISA−	ELISA+		Odds Ratio	Fisher Test *p*
	Frequency N = 87 (%)	Frequency N = 16 (%)	95% CI		
Abdominal pain in the last 3 months	66 (75.9)	11 (68.8)	44.4–85.8	0.700	0.543
Weight loss in the last 3 months	47 (54.0)	9 (56.2)	33.2–76.9	1.094	1.000
Diarrhea in the last 3 months	17 (19.5)	5 (31.2)	14.2–55.6	1.872	0.325
Headache	8 (9.2)	1 (6.2)	1.1–28.3	0.658	1.000
Nausea	6 (6.9)	2 (12.5)	3.5–36.0	1.929	0.607
Vomiting	5 (5.7)	3 (18.8)	6.6–43.0	3.785	0.106
Fever	5 (5.7)	2 (12.5)	3.5–36.0	2.343	0.297
Abdominal pain	2 (2.3)	1 (6.2)	1.1–28.3	2.833	0.401

**Table 4:** Comparison between gut microbiota composition and *F. hepatica* infection in school-aged children from San Pablo, Cajamarca, Peru.

Bacterium	ELISA−	ELISA+		Fisher Test *p*
	Frequency N = 87 (%)	Frequency N = 16 (%)	95% CI	
*Prevotella*	87 (100.0)	15 (93.8)	71.1–98.9	0.155
*Firmicutes*	85 (97.7)	16 (100.0)	80.6–100.0	1.000
*Bacteroidetes*	85 (97.7)	15 (93.8)	71.1–98.9	0.401
*Eubacterium*	78 (89.7)	15 (93.8)	71.7–98.9	1.000
*Proteobacteria*	75 (86.2)	11 (68.8)	44.4–85.8	0.135
*Lactobacillus*	63 (72.4)	6 (37.5)	18.5–61.4	0.010
*Bacteroides*	61 (70.1)	6 (37.5)	18.5–61.4	0.020
*Clostridium*	60 (69.0)	2 (12.5)	3.5–36.0	<0.001
*Enterococcus*	53 (60.9)	8 (50.0)	2.8–72.0	0.423
*Bifidobacterium*	32 (36.8)	1 (6.2)	1.1–28.3	0.018
*Actinobacteria*	20 (23.0)	4 (25.0)	10.2–49.5	1.000
*Veillonella*	5 (5.8)	0 (0.0)	0.0–19.4	1.000
*Fusobacterium*	5 (5.8)	0 (0.0)	0.0–19.4	1.000

## Data Availability

Data are contained within the article.

## References

[1] Cwiklinski K., O’Neill S.M., Donnelly S., Dalton J.P. (2016). A prospective view of animal and human Fasciolosis. Parasite Immunol..

[2] Mas-Coma S., Bargues M.D., Valero M.A. (2018). Human fascioliasis infection sources, their diversity, incidence factors, analytical ethods and prevention measures. Parasitology.

[3] Nyindo M., Lukambagire A.H. (2015). Fascioliasis: An Ongoing Zoonotic Trematode Infection. Biomed. Res. Int..

[4] Marcos L., Terashima Leguia G., Canales M., Espinoza J.R., Gotuzzo E. (2007). La Infección por *Fasciola hepática* en el Perú: Una Enfermedad Emergente. Rev. Gastroenterol. Perú.

[5] Espinoza J., Terashima A., Herrera-Velit P., Marcos L.A. (2010). Fasciolosis humana y animal en el Perú: Impacto en la economía de las zonas endémicas. Rev. Peru Med. Exp. Salud Publica.

[6] Gironènes N., Valero M.A., García-Bodelón M.A., Chico-Calero I., Punzón C., Presno M., Mas-Coma S. (2007). Immune suppression in advanced chronic fascioliasis: An experimental study in a rat model. J. Infect. Dis..

[7] Dalton J.P., Robinson M.W., Mulcahy G., O’Neill S.M., Donnelly S. (2013). Immunomodulatory molecules of *Fasciola hepatica*: Candidates for both vaccine and immunotherapeutic development. Vet. Parasitol..

[8] (2013). Drugs for Parasitic Infections.

[9] Villegas F., Angles R., Barrientos R., Barrios G., Valero M.A., Hamed K., Grueninger H., Ault S.K., Montresor A., Engels D. (2012). Administration of triclabendazole is safe and effective in controlling fascioliasis in an endemic community of the Bolivian Altiplano. PLoS Negl. Trop. Dis..

[10] Sezgin O., Altintas E., Disibeyaz S., Saritas Ü., Sahin B. (2004). Hepatobiliary fascioliasis: Clinical and radiologic features and endoscopic management. J. Clin. Gastroenterol..

[11] Gandhi P., Schmitt E.K., Chen C.W., Samantray S., Venishetty V.K., Hughes D. (2019). Triclabendazole in the treatment of human fascioliasis: A review. Trans. R. Soc. Trop. Med. Hyg..

[12] Clemente J.C., Ursell L.K., Parfrey L.W., Knight R. (2012). The Impact of the Gut Microbiota on Human Health: An Integrative View. Cell.

[13] Toro-Londono M.A., Bedoya-Urrego K., Garcia-Montoya G.M., Galvan-Diaz A.L., Alzate J.F. (2019). Intestinal parasitic infection alters bacterial gut microbiota in children. PeerJ.

[14] Wang B., Yao M., Lv L., Ling Z., Li L. (2017). The Human Microbiota in Health and Disease. Engineering.

[15] Mutapi F. (2015). The gut microbiome in the helminth infected host. Trends Parasitol..

[16] Glendinning L., Nausch N., Free A., Taylor D.W., Mutapi F. (2014). The microbiota and helminths: Sharing the same niche in the human host. Parasitology.

[17] Leung J.M., Graham A.L., Knowles S.C.L. (2018). Parasite-Microbiota Interactions With the Vertebrate Gut: Synthesis Through an Ecological Lens. Front. Microbiol..

[18] Houlden A., Hayes K.S., Bancroft A.J., Worthington J.J., Wang P., Grencis R.K., Roberts I.S. (2015). Chronic *Trichuris muris* Infection in C57BL/6 Mice Causes Significant Changes in Host Microbiota and Metabolome: Effects Reversed by Pathogen Clearance. PLoS ONE.

[19] Wu S., Li R.W., Li W., Beshah E., Dawson H.D., Urban J.F. (2012). Worm Burden-Dependent Disruption of the Porcine Colon Microbiota by *Trichuris suis* Infection. PLoS ONE.

[20] Nourrisson C., Scanzi J., Pereira B., NkoudMongo C., Wawrzyniak I., Cian A., Viscogliosi E., Livrelli V., Delbac F., Dapoigny M. (2014). *Blastocystis* Is Associated with Decrease of Fecal Microbiota Protective Bacteria: Comparative Analysis between Patients with Irritable Bowel Syndrome and Control Subjects. PLoS ONE.

[21] Kay G.L., Millard A., Sergeant M.J., Midzi N., Gwisai R., Mduluza T., Ivens A., Nausch N., Mutapi F., Pallen M. (2015). Differences in the faecal microbiome in *Schistosoma haematobium* infected children vs. uninfected children. PLoS Negl. Trop. Dis..

[22] Lee S.C., Tang M.S., Lim Y.A., Choy S.H., Kurtz Z.D., Cox L.M., Gundra U.M., Cho I., Bonneau R., Blaser M.J. (2014). Helminth colonization is associated with increased diversity of the gut microbiota. PLoS Negl. Trop. Dis..

[23] Partida-Rodríguez O., Serrano-Vázquez A., Nieves-Ramírez M.E., Moran P., Rojas L., Portillo T., González E., Hernández E., Finlay B.B., Ximenez C. (2017). Human Intestinal Microbiota: Interaction between Parasites and the Host Immune Response. Arch. Med. Res..

[24] Valero M.A., Periago M.V., Pérez-Crespo I., Angles R., Villegas F., Aguirre C., Strauss W., Espinoza J.R., Herrera P., Terashima A. (2012). Field evaluation of a coproantigen detection test for fascioliasis diagnosis and surveillance in human hyperendemic areas of Andean countries. PLoS Negl. Trop. Dis..

[25] Murri M., Leiva I., Gomez-Zumaquero J.M., Tinahones F.J., Cardona F., Soriguer F., Queipo-Ortuño M.I. (2013). Gut microbiota in children with type 1 diabetes difers from that in healthy children: A case–control study. BMC Med..

[26] Benavides-Ward A., Vasquez-Achaya F., Silva-Caso W., Aguilar-Luis M.A., Mazulis F., Urteaga N., del Valle-Mendoza J. (2018). *Helicobacter pylori* and its relationship with variations of gut microbiota in asymptomatic children between 6 and 12 years. BMC Res. Notes..

[27] Marcos L., Maco V., Samalvides F., Terashima A., Espinoza J.R., Gotuzzo E. (2006). Risk factors for *Fasciola hepatica* infection in children: A case-control study. Trans. R. Soc. Trop. Med. Hyg..

[28] McSorley H.J., Hewitson J.P., Maizels R.M. (2013). Immunomodulation by helminth parasites: Defining mechanisms and mediators. Int. J. Parasitol..

[29] Alvarado R., To J., Lund M.E., Pinar A., Mansell A., Robinson M.W., O’Brien B.A., Dalton J.P., Donnelly S. (2017). The immune modulatory peptide FhHDM-1 secreted by the helminth *Fasciola hepatica* prevents NLRP3 inflammasome activation by inhibiting endolysosomal acidification in macrophages. FASEB J..

[30] Begley M., Gahan C.G., Hill C. (2005). The interaction between bacteria and bile. FEMS Microbiol. Rev..

[31] Schnabl B., Brenner D.A. (2014). Interactions between the intestinal microbiome and liver diseases. Gastroenterology.

[32] Henao-Mejia J., Elinav E., Thaiss C.A., Licona-Limon P., Flavell R.A. (2013). Role of the intes-tinal microbiome in liver disease. J. Autoimmun..

[33] Schnabl B. (2013). Linking intestinal homeostasis and liver disease. Curr. Opin. Gastroenterol..

[34] Chassaing B., Etienne-Mesmin L., Gewirtz A.T. (2014). Microbiota-liver axis in hepatic dis-ease. Hepatology.

[35] Nguyen Thu H., Dermauw V., Tran Huy T., Roucher C., Dorny P., Nguyen Thi H., Trung K.H., Dao Van T., Do Nhu B., Nguyen Kim T. (2022). Diagnosing Human Fascioliasis Using ELISA Immunoassays at a Tertiary Referral Hospital in Hanoi: A Cross-Sectional Study. Trop. Med. Infect. Dis..

[36] Harinasuta T., Pungpak S., Keystone J.S. (1993). Trematode infections. Opisthorchiasis, clonorchiasis, fascioliasis, and paragonimiasis. Infect. Dis. Clin. N. Am..

[37] Cruz YLópez O.R., Gómez de la Vega E., Cárdenas-Perea M.E., Gutiérrez-Dávila A., Tamariz-Cruz O.J. (2016). Human fasciolosis diagnosed in the acute phase: A first clinical report in Mexico. Rev. Gastroenterol. Mex..

[38] Sezgın O., Altintaş E., Tombak A., Uçbılek E. (2010). *Fasciola hepatica*-induced acute pancreatitis: Report of two cases and review of the literature. Turk. J. Gastroenterol..

[39] Kaya M., Beştaş R., Cetin S. (2011). Clinical presentation and management of *Fasciola hepatica* infection: Single-center experience. World J. Gastroenterol..

[40] Lin L., Zhang J. (2017). Role of intestinal microbiota and metabolites on gut homeostasis and human diseases. BMC Immunol..

[41] Wexler A.G., Goodman A.L. (2017). An insider’s perspective: Bacteroides as a window into the microbiome. Nat. Microbiol..

[42] Wexler H.M. (2007). Bacteroides: The good, the bad, and the nitty-gritty. Clin. Microbiol. Rev..

[43] Azad M.A., Sarker M., Li T., Yin J. (2018). Probiotic Species in the Modulation of Gut Microbiota: An Overview. Biomed. Res. Int..

[44] Bron P.A., Van Baarlen P., Kleerebezem M. (2012). Emerging molecular insights into the interaction between probiotics and the host intestinal mucosa. Nat. Rev. Microbiol..

[45] Lopetuso L.R., Scaldaferri F., Petito V., Gasbarrini A. (2013). Commensal Clostridia: Leading players in the maintenance of gut homeostasis. Gut Pathog..

[46] Segain J.P., Raingeard de la Blétière D., Bourreille A., Leray V., Gervois N., Rosales C., Ferrier L.,  Bonnet C., Blottière H.M., Galmiche J.P.I. (2000). Butyrate inhibits inflammatory responses through NFkappaB inhibition: Implications for Crohn’s disease. Gut.

[47] Hague A., Elder D.J., Hicks D.J., Paraskeva C. (1995). Apoptosis in colorectal tumour cells: Induction by the short chain fatty acids butyrate, propionate and acetate and by the bile salt deoxycholate. Int. J. Cancer.

[48] Ramírez A.L., Herrera G., Muñoz M., Vega L., Cruz-Saavedra L., García-Corredor D., Pulido-Medellín M., Bulla-Castañeda D.M., Giraldo J.C., Bernal M.C. (2021). Describing the intestinal microbiota of Holstein Fasciola-positive and -negative cattle from a hyperendemic area of fascioliasis in cen-tral Colombia. PLoS Negl. Trop. Dis..

[49] Ianiro G., Iorio A., Porcari S., Masucci L., Sanguinetti M., Perno C.F., Gasbarrini A., Putignani L., Cammarota G. (2022). How the gut parasitome affects human health. Therap. Adv. Gastroenterol..

[50] Gurung M., Li Z., You H., Rodrigues R., Jump D.B., Morgun A., Shulzhenko N. (2020). Role of gut microbiota in type 2 diabetes pathophysiology. EBioMedicine.

[51] Beyhan Y.E., Yıldız M.R. (2023). Microbiota and parasite relationship. Diagn. Microbiol. Infect. Dis..

